# Interleukin-13 Promotes Susceptibility to Chlamydial Infection of the Respiratory and Genital Tracts

**DOI:** 10.1371/journal.ppat.1001339

**Published:** 2011-05-05

**Authors:** Kelly L. Asquith, Jay C. Horvat, Gerard E. Kaiko, Alison J. Carey, Kenneth W. Beagley, Philip M. Hansbro, Paul S. Foster

**Affiliations:** 1 Centre for Asthma and Respiratory Disease and Hunter Medical Research Institute, University of Newcastle, Newcastle, Australia; 2 Institute of Health and Biomedical Innovation, Queensland University of Technology, Kelvin Grove, Australia; Yale University School of Medicine, United States of America

## Abstract

*Chlamydiae* are intracellular bacteria that commonly cause infections of the respiratory and genital tracts, which are major clinical problems. Infections are also linked to the aetiology of diseases such as asthma, emphysema and heart disease. The clinical management of infection is problematic and antibiotic resistance is emerging. Increased understanding of immune processes that are involved in both clearance and immunopathology of chlamydial infection is critical for the development of improved treatment strategies. Here, we show that IL-13 was produced in the lungs of mice rapidly after *Chlamydia muridarum* (*Cmu*) infection and promoted susceptibility to infection. Wild-type (WT) mice had increased disease severity, bacterial load and associated inflammation compared to IL-13 deficient (−/−) mice as early as 3 days post infection (p.i.). Intratracheal instillation of IL-13 enhanced bacterial load in IL-13−/− mice. There were no differences in early IFN-g and IL-10 expression between WT and IL-13−/− mice and depletion of CD4+ T cells did not affect infection in IL-13−/− mice. Collectively, these data demonstrate a lack of CD4+ T cell involvement and a novel role for IL-13 in innate responses to infection. We also showed that IL-13 deficiency increased macrophage uptake of *Cmu in vitro* and *in vivo*. Moreover, the depletion of IL-13 during infection of lung epithelial cells *in vitro* decreased the percentage of infected cells and reduced bacterial growth. Our results suggest that enhanced IL-13 responses in the airways, such as that found in asthmatics, may promote susceptibility to chlamydial lung infection. Importantly the role of IL-13 in regulating infection was not limited to the lung as we showed that IL-13 also promoted susceptibility to *Cmu* genital tract infection. Collectively our findings demonstrate that innate IL-13 release promotes infection that results in enhanced inflammation and have broad implications for the treatment of chlamydial infections and IL-13-associated diseases.

## Author Summary

Chlamydial infections are a common cause of respiratory, genital tract and eye diseases, and infections are clinically associated with the aetiology of asthma, emphysema, heart disease and Alzheimer's. However, it is not known what immune factors regulate enhanced susceptibility to infection and immunopathology. In this study we have investigated the role of the immune factor, interleukin-13 (IL-13), in *C. muridarum* infections in mice. IL-13 is produced rapidly after respiratory infection in normal mice. However, mice deficient in IL-13 have reduced clinical symptoms and numbers of *C. muridarum* in their lungs after infection. The immune cells of mice deficient in IL-13 phagocytose more *C. muridarum* and their lung cells have less infection. The role of IL-13 is not restricted to the lung as IL-13-deficient mice have significantly lower levels of bacterial replication and more mild disease during genital tract infection. Our results suggest that IL-13 responses enhance chlamydial infections and that this factor may be a new therapeutic target for the treatment of disease.

## Introduction

*Chlamydiae* are Gram-negative, obligate intracellular bacteria that commonly cause respiratory and genital tract as well as ocular infections in humans. Globally, *Chlamydophila pneumoniae* has been estimated to account for 5% of cases of bronchitis and sinusitis, and up to 22% of cases of community-acquired pneumonia requiring hospitalisation [1], [2]. *Chlamydia trachomatis* is the world's most common sexually transmitted bacterial infection with an estimated 92 million new cases reported annually [3], and vertical transmission of *C. trachomatis* can initiate eye infections and pneumonia in new-borns [4], [5]. *Chlamydiae* commonly cause asymptomatic infections and significantly, between 50–80% and 10–20% of adults have anti-*C. pneumoniae* and anti-*C. trachomatis* antibodies respectively, indicating the high prevalence of these infections within the community [1], [6], [7], [8]. Furthermore, chlamydial infection has been linked with a number of chronic disease states including asthma [9], chronic obstructive pulmonary disease (COPD) [10], [11], atherosclerotic cardiovascular disease [12], and neurodegenerative disorders such as Alzheimer's disease [13]. Understanding the complex immunobiology of host-pathogen interactions and the delineation of the specific responses that drive clearance *versus* tissue damage are of paramount importance for the prevention and treatment of chlamydial infection and diseases.

CD4+ T helper type 1 (Th1) cells secreting IFN-γ play critical roles in the clearance of infection. The rate of clearance of *Chlamydia* from infected mouse lungs is directly proportional to increases in IFN-γ levels [14], [15], [16], and the absence of this cytokine or its receptor drives infection into a persistent state [17], [18], [19]. IFN-γ enhances the ability of macrophages to clear chlamydial infections [20], [21] and it is a powerful activator of indoleamine 2,3-dioxygenase (IDO) and inducible nitric oxide sythase (iNOS), which prevent bacterial growth by limiting tryptophan availability and upregulating nitric oxide (NO) production, respectively [22], [23], [24], [25]. Although *Chlamydiae* predominantly colonise epithelial cells, new evidence suggests that they can also infect smooth muscle cells, vascular endothelial cells and components of the immune system including macrophages and dendritic cells [26], [27]. Importantly, infection of alveolar macrophages of both human and mouse origin has been demonstrated and is associated with enhanced production of anti-inflammatory cytokines [28], [29], [30].

Severe forms of tissue damage due to *Chlamydia* infections of the respiratory and genital tracts are generally caused by infections that elicit both cytopathic and delayed type hypersensitivity immunopathologic destruction of the epithelium [31], [32]. In the case of *C. pneumoniae* and *C. trachomatis* this can lead to pneumonia and pelvic inflammatory disease and infertility, respectively. At the cell and molecular level immunopathology may consist of excessive infiltration of neutrophils, inflammatory monocytes, and the over-expression of the pro-inflammatory cytokines IL-1β and TNF-α [33], [34], [35].

Studies in animals have shown that a deficiency in the IFN-γ response, an overtly suppressive IL-10 response, or a delay in the development of a global T cell response during chlamydial infection can all lead to enhanced bacterial dissemination and disease sequelae [15], [16], [36]. Given these previous studies, it seems plausible that the development of anti-chlamydial Th2 immune responses could lead to increased disease susceptibility and immunopathology. In support of this concept, studies in our laboratory have shown that pulmonary infection with the natural mouse pathogen *C. muridarum* (Cmu) can lead to enhanced production of the Th2 cytokine IL-13. Cmu infection early in life leads to increased production of IL-13 following allergen challenge in adult mice [37], [38]. On a cellular level, Cmu infection of bone marrow-derived dendritic cells (BMDC) modulates the cytokine profile from both DCs and T cells to produce increased IL-13 *in vitro* [26]. Furthermore, urogenital *Cmu* infection in MyD88-deficient mice leads to a dominant Th2 response, skewed from the Th1/Th17 response of WT mice, which is associated with an ascending *Cmu* infection and severe pathology in the upper genital tract [39]. Allergic asthma is characterised by the infiltration of CD4+ Th type 2 (Th2) cells, which produce a specific subset of cytokines (e.g. IL-4, IL-5, IL-10 and IL-13) which have been linked to the pathogenesis of disease [40]. Clinical evidence directly links *C. pneumoniae* infection to asthma [41], [42], [43], [44]. However, the mechanistic basis underlying this relationship remains poorly understood. Enhanced production of IL-13 in response to chlamydial infection may contribute to the induction and exacerbation of asthma [26], [37], [38].

IL-13 has been described as a susceptibility factor for infection with both *Leishmania major* and *Crytococcus neoformans* [45], [46]. Both of these studies focus on the adaptive immune response to infection and attribute the ratio of Th2 cell derived IL-13 to Th1 cell IFN-γ as a key factor in determining the ability to mount a protective immune response and elimination of the pathogen. IL-13 is also produced by innate immune cells but the role of this cytokine in innate host defence against infection has received little attention. There is the potential that *Chlamydia* may induce innate and/or adaptive IL-13 responses to promote infection, which has significant implications for chlamydial respiratory and genital tract diseases, and associated conditions. In the present study we demonstrate that early production of IL-13 during the innate immune response plays a critical and previously unrecognised role in promoting *Cmu* infection of the respiratory and genital tract.

## Results

### Absence of IL-13 reduces susceptibility to and improves clearance of *Cmu* lung infection

**Figure 1:**
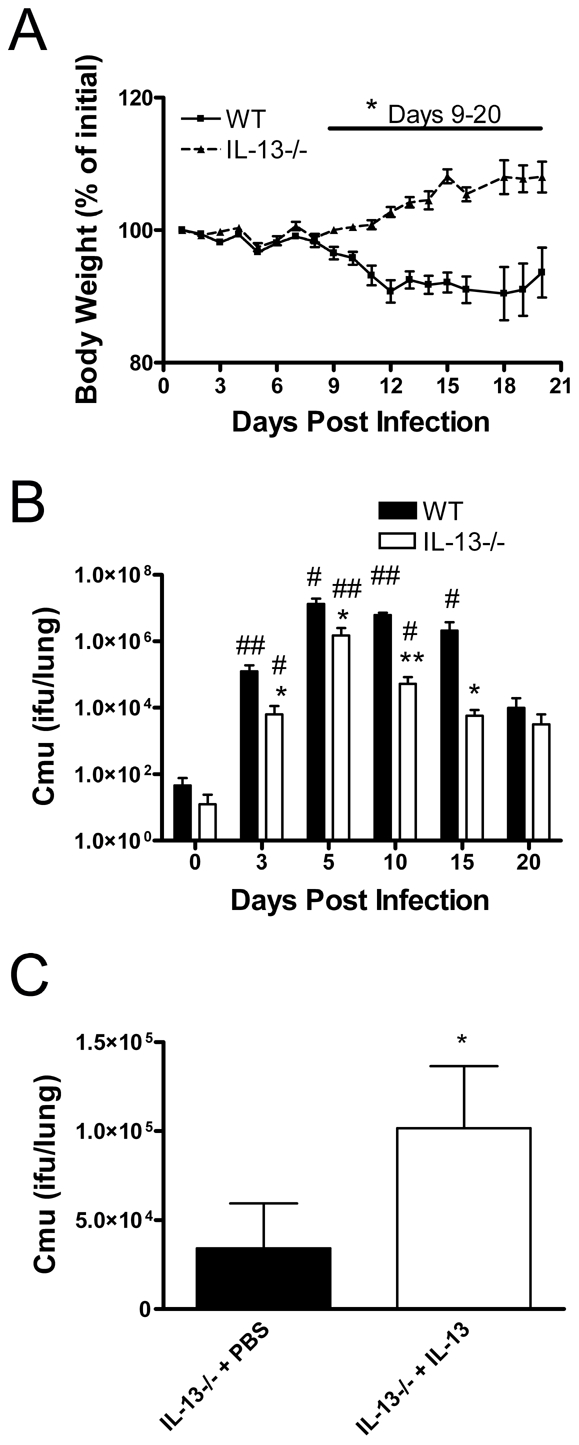
IL-13 deficiency reduces disease severity and enhances bacterial clearance during *Chlamydia muridarum* (*Cmu*) lung infection. WT and IL-13−/− mice were infected i.n. with 100 ifu of *Cmu* and monitored for 20 days post infection. (A) Percentage body weight change from pre-infection weight was monitored and (B) *Cmu* numbers in the lungs were determined by quantitative real-time PCR (qPCR). IL-13−/− mice were intratracheally treated with recombinant mouse (rm)IL-13 or PBS prior to *Cmu* infection and (C) *Cmu* numbers in the lungs were determined by qPCR on day 5 p.i. Results are presented as mean ± SEM and significant differences between WT and IL-13−/− mice or rmIL-13 and PBS-treated IL-13−/− mice are shown as *p<0.05 and **p<0.01. Significant differences in chlamydial numbers from time 0 of the same strain are represented by #p<0.05 and ##p<0.01.

We first assessed the role of IL-13 in chlamydial respiratory infection. Adult WT and IL-13−/− mice were infected intranasally (i.n.) with *Cmu* and disease severity and bacterial numbers in the lungs were determined over time. Weight is an established indicator of disease severity, and substantial weight loss was observed in WT mice from 7 days after infection (Figure 1A). At this stage of infection mice begin to display significant histopathological changes in the lung [37]. By contrast, IL-13−/− mice increased in body weight from day 7 p.i. (Figure 1A). These differences in weight change between WT and IL-13−/− mice were significant from 9-20 days after infection (Figure 1A). Differences in weight changes between WT and IL-13−/− mice also correlated with bacterial load in the lungs. WT mice had a significant increase in bacterial load from day 3 to day 15 p.i. (Figure 1B). By contrast, IL-13−/− mice had significantly lower levels of *Cmu* at day 3 p.i. and throughout the course of infection (Figure 1B). The administration of recombinant mouse (rm)IL-13 to the lungs of IL-13−/− mice prior to *Cmu* lung infection resulted in a significant increase in bacterial load at day 5 p.i. compared to untreated controls (Figure 1C). Together, these data demonstrate that the early presence of IL-13 during the innate host defence response plays a central role in promoting susceptibility to *Cmu* lung infection. Furthermore IL-13 deficiency suppresses the development of clinical signs infection as a result of an enhanced ability to clear the bacteria.

### Enhanced *Cmu* clearance in the absence of IL-13 is associated with decreased airway inflammation

**Figure 2:**
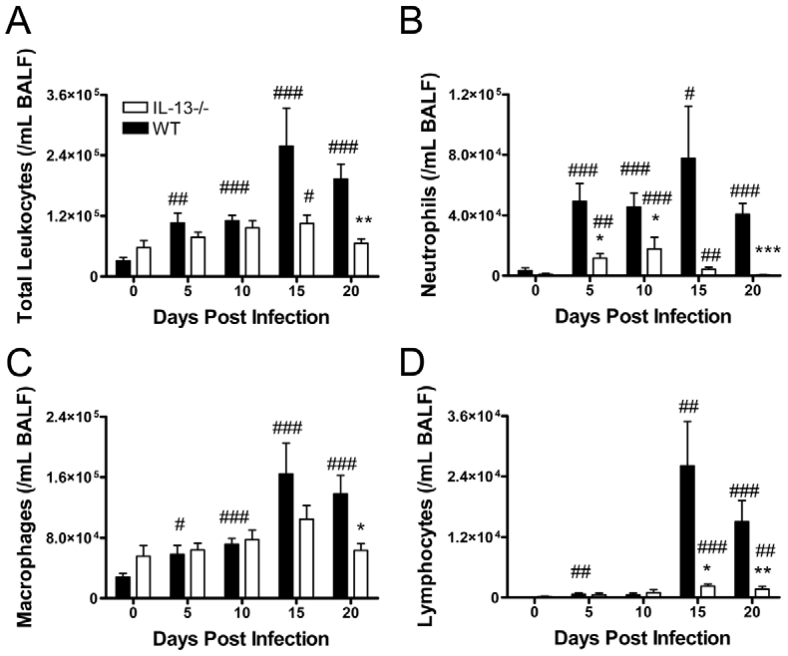
IL-13 deficiency reduces airway inflammation during *Chlamydia muridarum* (*Cmu*) lung infection. WT and IL-13−/− mice were infected i.n. with 100 ifu of *Cmu* and (A) total leukocyte, (B) neutrophil, (C) macrophage and (D) lymphocyte numbers in bronchoalveolar lavage fluid (BALF) were determined for 20 days after infection. Eosinophils were not detected in any groups. Results are presented as mean ± SEM and significant differences between WT and IL-13−/− mice are shown as *p<0.05, **p<0.01 and ***p<0.001. Significant differences from time 0 of the same strain are represented by #p<0.05, ##p<0.01 and ###p<0.001.

We then characterised the influence of IL-13 on inflammatory responses to *Cmu* infection. Pulmonary inflammation was assessed by enumerating leukocytes in the bronchoalveolar lavage fluid (BALF) of infected mice. Infection of WT mice led to a significant increase in the total number of leukocytes, neutrophils and macrophages present in the airways from as early as 5 days p.i. and significant increases in lymphocyte numbers were observed from 15 days p.i. (Figure 2A–D). IL-13 deficiency resulted in a significant reduction in total leukocyte, neutrophil, macrophage and lymphocyte influx into the lungs compared to WT controls, with limited evidence of increases in cellular infiltrates compared to baseline levels. (Figure 2A–D). Eosinophils were not detected in the airways of infected mice (not shown). Differences in neutrophil influx between strains were detected at 5 days p.i., whereas significant differences in macrophage and lymphocyte numbers were not apparent until the later stages of infection. An increased level of blood neutrophilia was also observed in infected WT compared to IL-13−/− mice (data not shown). Therefore, IL-13 deficiency results in not only reduced bacterial load but decreases in associated inflammation.

### IL-13 is expressed in the lungs during infection and influences the expression of cytokines important in pathogen clearance

**Figure 3:**
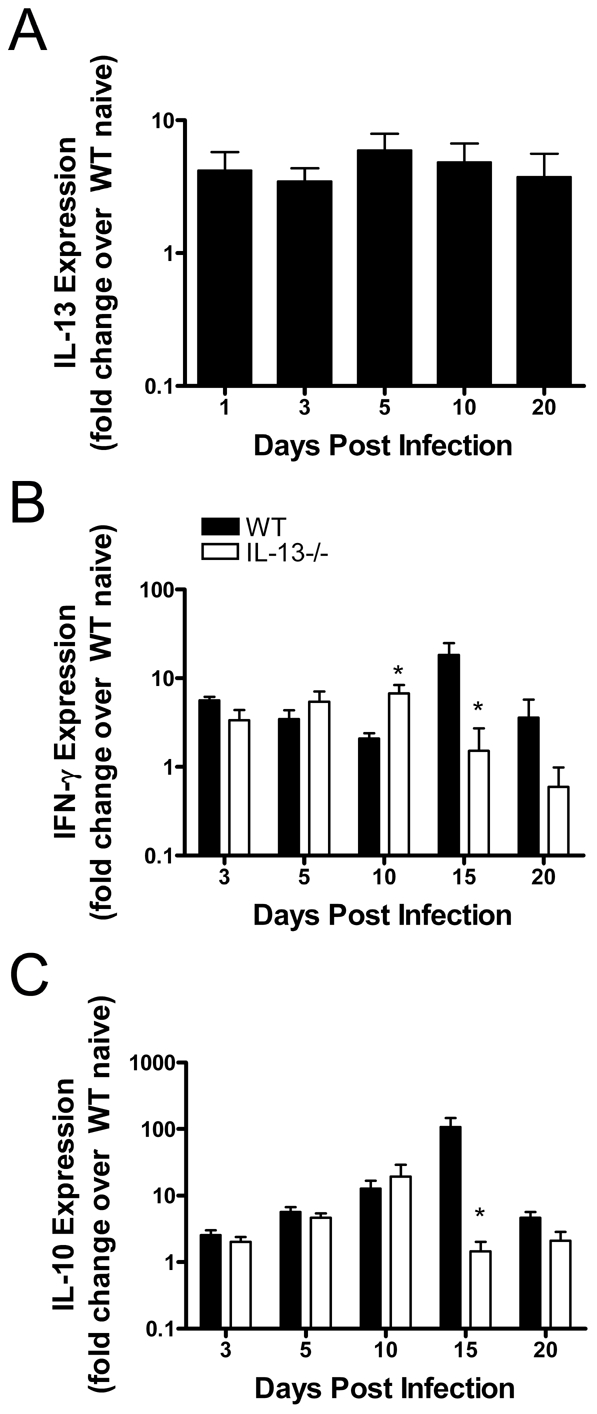
IL-13 expressed in the WT lung after *Chlamydia muridarum* (*Cmu)* infection modulates cytokines important in pathogen clearance. WT and IL-13−/− mice were infected i.n. with 100 ifu of *Cmu*. Expression of (A) IL-13 (WT only), (B) IFN-γ and (C) IL-10 was measured in RNA extracted from whole lung tissue by quantitative real-time PCR. Results are expressed as fold change over WT naive control mice and are presented as mean ± SEM. Significant differences between strains are shown as *p<0.05.

Next we assessed the temporal expression of IL-13 during chlamydial infection. Quantitative real-time PCR of lung tissue RNA was used to measure the expression of IL-13 in WT mice following i.n. infection and normalised to uninfected controls. Infection was accompanied by a 5-fold increase in IL-13 expression, which was observed as early as 24 hours p.i. compared to naïve controls (Figure 3A). This increased level of IL-13 expression in the lungs of infected mice was maintained for at least 20 days p.i when compared to IL-13 expression in naïve tissue. These data represent the first report of pulmonary IL-13 expression during respiratory chlamydial infection.

To explore the mechanisms by which IL-13 mediates the host response to *Cmu* lung infection, we determined whether IL-13 influences the expression of IFN-γ and IL-10, factors known to play a central role in the immune response to chlamydial infection. Notably, the expression of IFN-γ and IL-10 were not different between naïve WT and IL-13−/− mice and the onset of IFN-γ and IL-10 production was not affected by the absence of IL-13 during the early stages of infection. Indeed, on days 3 and 5 p.i. there were no significant differences between the expression of IFN-γ in infected IL-13−/− mice compared to WT controls (Figure 3B). On day 10, IFN-γ production in WT mice was less than IL-13−/− mice, however, by day 15 the production of IFN-γ was greater in WT mice. Interestingly, differences in the levels of IL-10 expression were not observed until day 15 p.i., where WT mice expressed more IL-10 compared to IL-13−/− mice (Figure 3C). These results demonstrate that IL-13 directly or indirectly influences the expression of other cytokines that have been implicated in host defence against chlamydial infection. However, since these changes were not observed until the later stages of infection they do not explain the effect of IL-13 deficiency on bacterial numbers during the early onset of *Cmu* infection. This indicates that there are other mechanisms rather than changes in cytokine responses or in T cell phenotype that result in reduced infection in the absence of IL-13.

### Enhanced clearance of *Cmu* respiratory infection in the absence of IL-13 is not dependent on CD4+ T-cells

**Figure 4:**
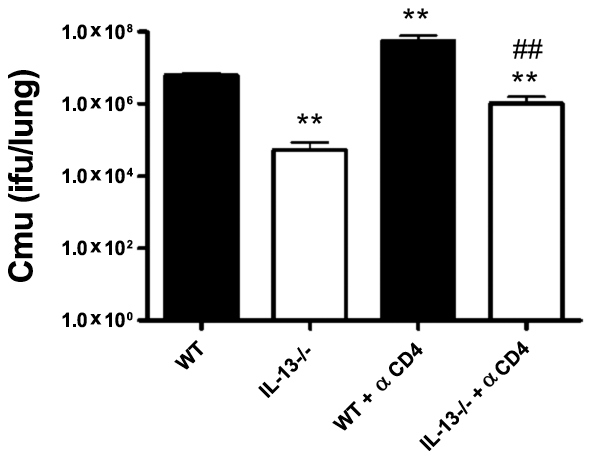
Enhanced clearance of *Chlamydia muridarum* (*Cmu*) infection in IL-13−/− mice is not dependent on CD4+T cells. WT and IL-13−/− mice were depleted of CD4+ T cells by treatment with anti-CD4 monoclonal antibody (αCD4) and infected i.n. with 100 ifu of *Cmu*. Controls were untreated WT and IL-13−/− mice that were infected with *Cmu*. *Cmu* numbers in the lung were determined at 10 days post infection by quantitative real-time PCR. Results are presented as mean ± SEM and significant differences compared to untreated WT are shown as **p<0.01 and compared to WT + αCD4 are shown as ##p<0.01.

The limited studies that have investigated the role of IL-13 in pathogen infection have focused on this molecule as a cytokine that is produced by activated CD4+ Th2 cells. By contrast, our data suggest that IL-13 plays an important role as early as 3 days p.i. (Figure 1B), and therefore is mediating innate rather than adaptive responses. To test this hypothesis we depleted CD4+ T cells using a specific monoclonal antibody (mAb) prior to and after i.n. infection of WT and IL-13−/− mice. FACS analysis confirmed that antibody treatment depleted CD4+ cell numbers in the lung to less than 5% of that in untreated WT mice (0.75 ± 0.11 Vs. 13.91 ± 1.26% viable cells, Figure S1). WT mice treated with anti-CD4 mAb had increased chlamydial load in the lungs compared to untreated controls (Figure 4). Both untreated and treated WT mice displayed a significant drop in body weight 10 days post infection (93.17 ± 1.49 and 94.83 ± 1.78% of initial weight, p<0.01, respectively), which became apparent earlier in the treated group (data not shown). By contrast, CD4+ cell depletion did not significantly affect bacterial load (Figure 4) or body weight in IL-13−/− mice (100.8 ± 0.70 and 100.57 ± 1.42% of initial weight in untreated and treated IL-13−/− mice respectively). Together, these observations suggest that the reduced susceptibility to chlamydial infection in the absence of IL-13 is not mediated by CD4+ T cells but is linked to the innate host defence response.

### Macrophages from IL-13 deficient mice show enhanced uptake of *Cmu in vitro* and *in vivo*

**Figure 5:**
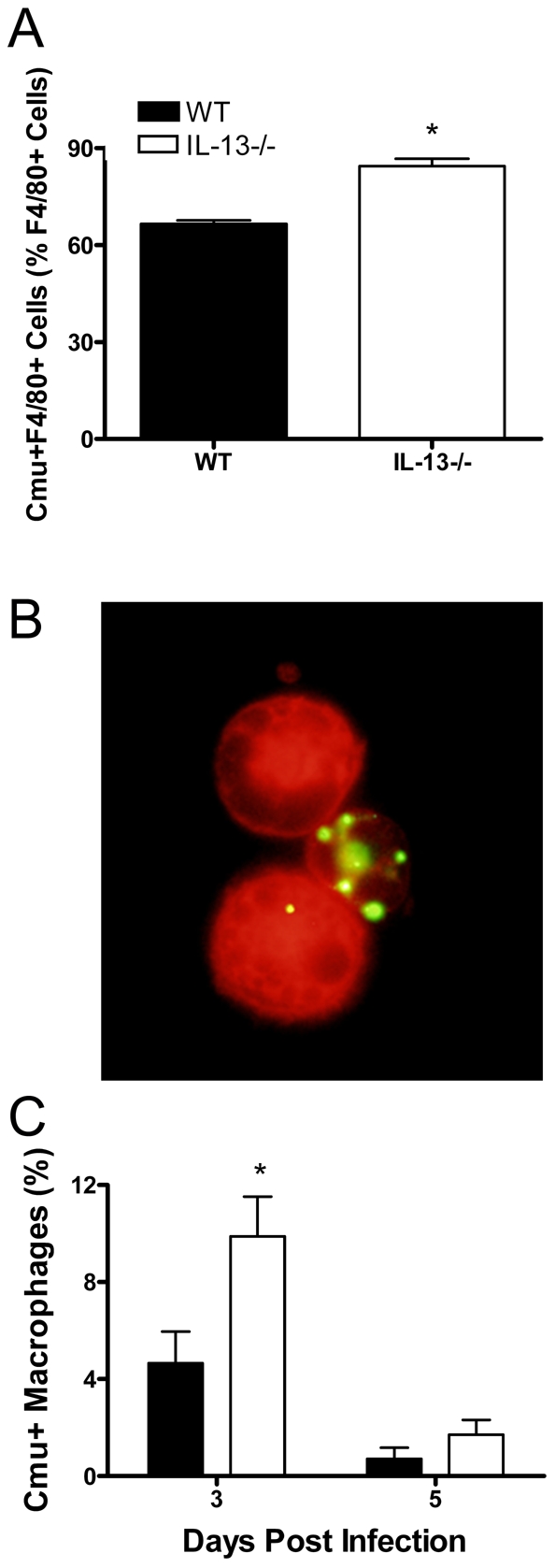
IL-13 deficiency enhances the uptake of *Chlamydia muridarum* (*Cmu*) by macrophages *in vitro* and *in vivo*. Bone marrow-derived (BM) macrophages were incubated with CFSE-labelled, UV-inactivated *Cmu*. (A) The percentage of macrophages (F4/80+ cells) containing *Cmu* (CFSE+F4/80+ cells) were determined using flow cytometry. WT and IL-13−/− mice were infected i.n. with 100 ifu *Cmu* and macrophages were isolated from bronchoalveolar lavage fluid at 3 and 5 days post infection. (B) Macrophages containing *Cmu* were identified using a *Chlamydia*-specific fluorescent labelling kit. (C) The percentage of macrophages that stained positive for *Cmu* was enumerated. Results are presented as mean ± SEM and significant differences between strains are shown as *p<0.05.

IL-13 is known to affect macrophage function and impair phagocytosis. Phagocytosis of bacteria by macrophages plays an important role in the innate defence against pathogens, including *Chlamydia*. Therefore, the effect of IL-13 deficiency on *Cmu* uptake by macrophages was investigated. Equal numbers of bone marrow-derived (BM) macrophages from WT and IL-13−/− mice were cultured in the presence of equal titres of UV-inactivated, CFSE-labelled *Cmu*. Cells were then washed to remove any free *Cmu* and the percentage of BM macrophages (F4/80+ cells) that had taken up *Cmu* (CFSE+) was determined using flow cytometry. The percentage of *Cmu* positive macrophages was significantly higher in cultures from IL-13−/− compared to WT mice (84.4 ± 2.3 Vs. 66.5 ± 1.1%, p<0.05, Figure 5A). Notably these methods directly measure phagocytosis rate of a specific number of *Cmu* by a specific number of macrophages and are not affected by the differing amounts of Cmu present in the two strains. To confirm these data *in vivo*, the effects of IL-13 on the function of macrophages during *Cmu* lung infection was also investigated. Alveolar macrophages were isolated from the BALF of infected WT and IL-13−/− mice and the engulfment of *Cmu* was assessed by staining them using a *Chlamydia*-specific fluorescent labelling kit (Figure 5B). Interestingly, only half as many BALF macrophages from WT mice stained positive for *Cmu* 3 days after infection compared to those from IL-13−/− mice (Figure 5C). This is despite total BALF macrophage numbers being similar (data not shown) and *Cmu* numbers increased in the lungs of WT compared to IL-13−/− mice at this stage of infection (Figure 1B). At 5 days after infection no strain-specific differences in the ability of macrophages to engulf *Cmu* were detected, however, this may be explained by the high numbers of *Cmu* observed in the lungs of WT mice at this stage of infection. Together, our findings show that in the absence of IL-13, the uptake of *Cmu* by macrophages is enhanced. Thus, impaired phagocytosis of *Cmu* may represent an important mechanism by which chlamydial clearance is delayed in the presence of IL-13.

### Depletion of IL-13 reduces the susceptibility of murine pulmonary epithelial cells to *Cmu* infection

**Figure 6:**
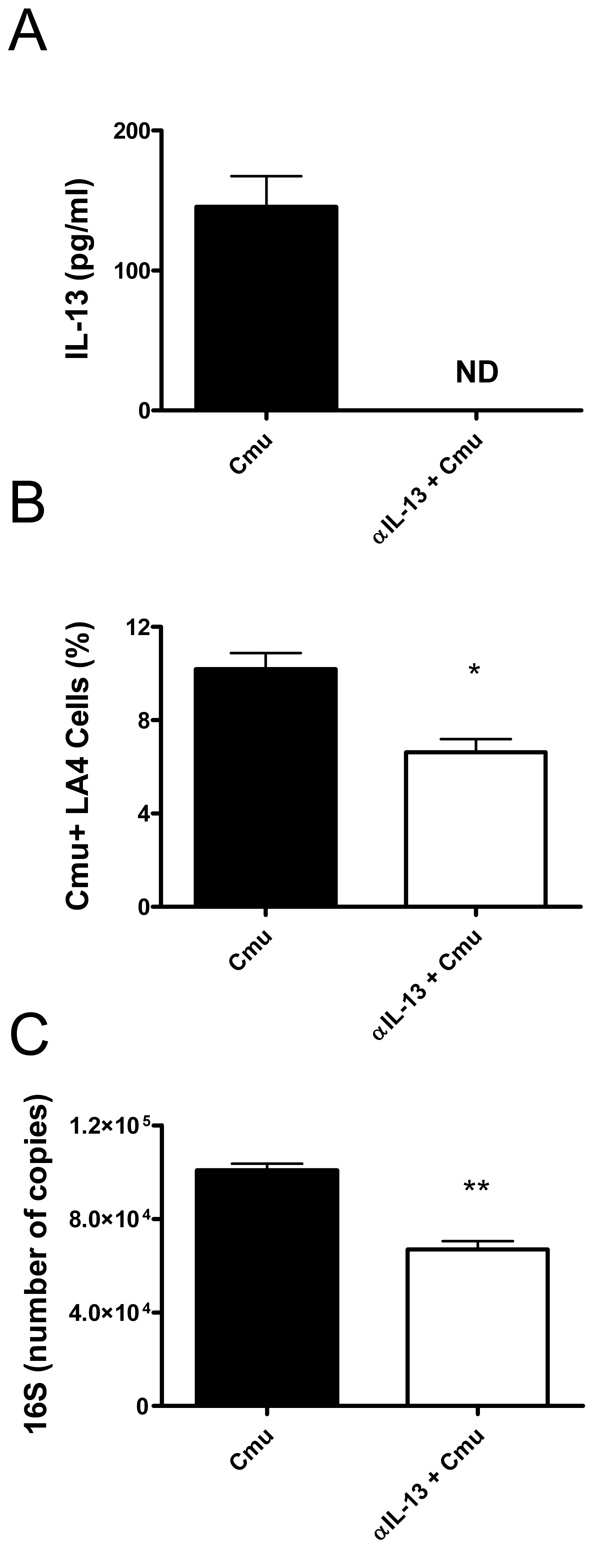
Depletion of IL-13 decreases the susceptibility of murine pulmonary epithelial cells to *Chlamydia muridarum* (*Cmu*) infection. Confluent LA4 cells were infected with *Cmu* and treated with 100 µg anti-IL-13 mAb (αIL-13 + *Cmu*) for 24 hours prior to and during infection. Control cells were infected with *Cmu* in the absence of mAb treatment. (A) Culture supernatants were collected at 24 hours post infection and IL-13 levels were determined by ELISA. (B) The percentage of infected cells (stained positive for *Cmu* inclusions) was determined using a *Chlamydia*-specific fluorescent labelling kit. (C) *Cmu* 16S RNA expression in infected cells was determined using quantitative real-time PCR. Results are presented as mean ± SEM. Significant differences between treated and untreated groups are shown as *p<0.05 and **p<0.01. ND  =  not detected.

The absence of IL-13 had profound effects during the early stages of infection. Therefore, we hypothesised that, in addition to influencing macrophage phagocytosis, IL-13 may affect the susceptibility of airway epithelial cells to infection and intracellular proliferation of *Cmu*. To investigate this hypothesis we established an *in vitro* model of infection with LA4 cells, an immortalised murine lung epithelial cell line. Confluent monolayers of LA4 cells were infected with *Cmu* and cells were treated with anti-IL-13 (αIL-13) mAb prior to and during infection. Infection was assessed by enumerating *Cmu* inclusion positive cells using fluorescent microscopy and by determining the levels of *Cmu* 16S RNA expression in cultures. 16S expression is an indicator of growth. Previous work in our laboratory has shown that *Cmu* infection of LA4 cells induces widespread cell lysis after 30 hours (not shown). Therefore, the effects of IL-13 depletion on *in vitro* infection were assessed after 24 hours. Incubation for 24 hours still allows for the formation of large inclusions within infected cells and is appropriate for analysing of the effect of IL-13 depletion on cellular susceptibility to chlamydial infection. αIL-13 mAb treatment depleted IL-13 protein levels 24 hours after infection (Figure 6A). Significantly, the depletion of IL-13 resulted in decreased susceptibility of LA4 cells to infection. αIL-13 treated LA4 cultures had lower percentages of *Cmu* inclusion positive cells 24 hours after infection compared to untreated controls (Figure 6B). This observation was confirmed by quantitative real-time PCR analysis, demonstrating that IL-13 deficient cultures had lower copies of *Cmu* 16S than untreated cultures (Figure 6C). These results indicate that IL-13 directly promotes infection of lung epithelial cells by *Cmu*.

### Absence of IL-13 reduces susceptibility to and improves clearance of chlamydial genital tract infection

**Figure 7:**
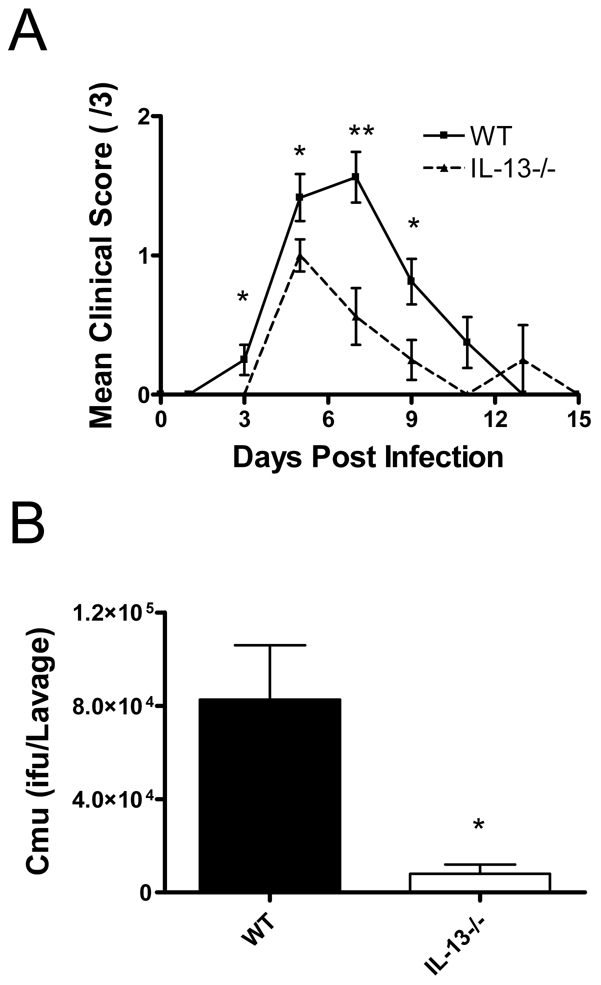
A role for IL-13 in *Chlamydia muridarum* (*Cmu*) genital tract infection. WT and IL-13−/− mice were infected intravaginally with 5×10^4^ ifu of *Cmu*. (A) *Cmu* numbers in vaginal lavage fluid at day 15 were measured by quantitative real-time PCR and (B) clinical score assessed according to the criteria listed in Table 1 for 15 days post infection. Results are presented as mean ± SEM and significant differences between strains are shown as *p<0.05 and **p<0.01.

To determine if IL-13 also played a role in promoting infection at other mucosal surfaces we intravaginally infected mice with *Cmu* and assessed disease severity and bacterial numbers over time. The chlamydial genital tract infection model employed in this study[47] has minimal effects on mouse body weight thus we used a clinical scoring system to determine disease severity (Table 1). Intravaginally infected WT mice had significantly higher clinical scores and more bacteria in vaginal lavage fluid 15 days p.i. compared to infected IL-13−/− mice (Figure 7). These data suggest that the role of IL-13 in host responses to *Chlamydia* infection is not restricted to the lung and suggests that this cytokine may play a role in other *Chlamydia* associated diseases.

**Table 1:** Clinical scoring of genital tract infections.

		Positive/Negative
A)	Vaginal mucus	
B)	Vaginal swelling and/or redness	
C)	Ruffled coat and/or hunching	
	Total score = A) + B) + C)	/3
Positive = 1, Negative = 0		

## Discussion

In this study we have shown for the first time that IL-13 responses to *Cmu* infection are important in establishing and promoting chlamydial infection, and inflammation and disease in the respiratory and genital tracts. These effects are associated with reduced macrophage phagocytosis and enhanced infection of airway epithelial cells. Chlamydial respiratory and genital tract infections and diseases are prevalent throughout the world and infections are associated with a number of other diseases, particularly asthma. Elucidating the mechanisms that determine susceptibility to infection and chlamydial clearance may identify new ways of treating these conditions. We have previously demonstrated that *Cmu* infection *in vitro* and *in vivo* induces IL-13 responses [26], [38] and that transfer of *Cmu* infected murine bone marrow dendritic cells (BMDCs) into recipient mice subverts the *in vivo* immune response from a protective Th1 to a non-protective Th2 phenotype that may promote chronic infection [26]. In the present study we extend these studies and show that IL-13 promotes chlamydial infection and has an unexpected role in the immediate host defence responses to infection. Importantly, this association appears to be at the level of the innate rather than the adaptive immune response and is not predicated on alterations in T cells responses or the concomitant suppression of IFNγ responses.

In our study IL-13 was increased 5-fold in the lung as early as 24 hours after infection. IL-13 is a potent cytokine and low levels in the airway enhances infection (Figure 1C) and induces profound changes in lung physiology [48]. WT mice displayed increased symptoms, *Cmu* load and airway inflammatory cell burden after infection compared to their IL-13−/− counterparts. Through their capacity to rapidly clear *Cmu* from the lungs IL-13−/− mice circumvent the development of sequelae associated with chronic infection, including the influx of inflammatory cells into the airway. The role of IL-13 in infection is typically attributed to its function as a Th2 cytokine, often acting as an immunological ‘switch’ by downregulating the Th1 response. Indeed IL-13 has been identified as a susceptibility factor for infection of mice by the protozoan parasite *Leishmania major* by suppressing the expression of IFNγ and IL-12 [45]. Moreover, over-expression of IL-13 in transgenic mice enhanced pulmonary infection of mice with *C. neoformans*, which was associated with increased Th2 cytokine production [46]. Interestingly, there was no inverse relationship detected between Th1 and Th2 cytokine production in this setting; however, increased fungal load correlated with attenuated Th17 cytokine production 60 days p.i.

In our study the influence of IL-13 on chlamydial infection was evident as early as 3 days p.i., again suggesting a novel role for this cytokine in the innate rather than acquired immune response to infection. The level of *Cmu* in the lungs of CD4-depleted, IL-13−/− mice was significantly lower than in both CD4-depleted, WT and untreated WT groups. Furthermore, there were no differences in IFN-g or IL-10 expression early in infection of WT and IL-13−/− mice. These results demonstrate that protection against infection in IL-13−/− mice is not dependent on CD4+ T-cell responses. These results confirm an important and central role for IL-13 in promoting infection during the early phases of the host defence response.

*Chlamydia* is capable of infecting a range of cell types, including alveolar macrophages and epithelial cells [27]. Our study demonstrates for the first time that the presence of IL-13 reduces the ability of macrophages to engulf *Cmu in vitro* and during *in vivo* lung infection. This may account for the enhanced ability of mice to clear this pathogen from the respiratory tract in the absence of this cytokine. Investigations of the role of IL-13 in immune responses to infection have shown that this molecule can induce the development of macrophages, which have a documented impairment in the ability to engulf and destroy intracellular pathogens *in vitro* [49], [50] and are associated with increased fungal burden in the lungs of mice during *C. neoformans* infection *in vivo* [46]. Interestingly, *in vitro* studies have also demonstrated that macrophage expression of the mannose receptor plays a pivotal role in determining susceptibility to chlamydial infection, although the significance of these findings *in vivo* have not yet been explored [51]. The capacity of IL-13 to modulate the innate immune response to infection in the present study may be underpinned by the development of macrophages with a reduced capacity to engulf and destroy *Chlamydia* in the lung.

It is also possible that IL-13 may condition the respiratory epithelium so that it is more susceptible to infection, thus the action of IL-13 is on structural cells as well as non-lymphoid cells that play key roles in host defence pathways. Furthermore, the fact that IL-13 mediates susceptibility to genital tract infection highlights the potential widespread role of this molecule in promoting chlamydial infection and diseases.

While CD4+ Th2 cells have typically been regarded as the principal source of IL-13, there is now a growing body of evidence that non-lymphoid cells are important producers of this cytokine and contribute to its associated pathologies. Mast cells [52], basophils [53], macrophages [54], bronchial mucosal cells [55], airway epithelial cells [56], dendritic cells [26] and natural killer T (NKT) cells [57] have all been demonstrated to generate IL-13. In our study IL-13 is produced rapidly, with an increase in expression apparent within 1 day p.i., which suggests that this cytokine is originating from non-lymphoid cells, with innate immune activity. Further studies are required to identify the key cell(s) that are the early cellular sources of IL-13 during *Cmu* infection.

The effects of IL-13 in promoting enhanced *Cmu* infection demonstrated in the current study may provide a basis for the widely observed clinical and experimental link between chlamydial infection and asthma [9], [38], [58], [59], [60]. Allergic airway inflammation in mice inhibits pulmonary host defence against other respiratory pathogens such as *Pseudomonas aeruginosa* [61] and alveolar macrophages from children with poorly controlled asthma have an impaired ability to phagocytose FITC-conjugated *Staphylococcus aureus* [62]. Furthermore BALB/c mice, which are biased towards Th2 cytokine responses, are markedly more susceptible to chlamydial lung infection than the Th1-predisposed C57BL/6 strain [16]. Increases in pulmonary IL-13 in asthmatic patients may promote susceptibility and contribute to the prevalence of chlamydial infection in these patient populations. Our *in vitro* evidence that IL-13 increases the susceptibility of airway epithelial cells to infection with *Cmu* also supports this concept.

In summary, our study reveals for the first time that production of IL-13 during the innate host defence phase plays a central role in establishing and promoting *Cmu* respiratory and genital tract infections. This role appears to be independent of CD4+ T cell-mediated adaptive immune responses and may be a result of the reduced ability of macrophages to engulf *Cmu* and an increased susceptibility of pulmonary epithelial cells to infection. This study enhances our understanding of the pathogenesis of chlamydial infection and identifies IL-13 as new potential target to attenuate infection, inflammation and pathology associated with *Chlamydia*.

## Materials and Methods

### Ethics statement

This study was carried out in strict accordance with the recommendations set out in the Australian code of practice for the care and use of animals for scientific purposes issued by the National Health and Medical Research Council (Australia). All protocols were approved by the University of Newcastle Animal Care and Ethics Committee and all efforts were made to minimise suffering.

### Mice

Adult (6–8 weeks old) WT BALB/c mice and IL-13−/− mice on a BALB/c background were supplied by the animal breeding facilities of the Australian National University or the University of Newcastle. Mice were housed under specific pathogen free conditions.

### Respiratory tract infection

Adult mice were infected i.n. with 100 ifu of Cmu (ATCC VR-123; in 30 µl of sucrose phosphate glutamate buffer [SPG])[37], [38], [60], [63]. Mice were monitored over a 20 day period and rate of weight gain/loss was used as a measure of clinical condition. At selected time points, mice were sacrificed by sodium pentobarbital overdose (Abbott Australasia, Kurnell, Australia) for analysis.

### Recombinant IL-13 administration

IL-13−/− mice were intratracheally administered rmIL-13 (10 ng, 30 µl of PBS, R&D Systems, Gymea, NSW) or PBS 6 hours prior to infection and sacrificed 5 days later for analysis.

### Genital tract infection

Adult mice were subcutaneously injected with 2 mg medroxyprogesterone acetate (Troy Laboratories, Smithfield, Australia) to synchronise their estrous cycles. Seven days later mice were infected intravaginally with 5×10^4^ ifu Cmu (in 20 µl SPG)[47]. Mice were monitored over a 15 day period and their clinical score determined according to specific signs of disease (Table 1). At selected time points, mice were sacrificed by sodium pentobarbital overdose for analysis.

### Assessment of bacterial levels

Whole lungs from mice infected i.n. with Cmu were removed and stored at −80°C. Vaginal lavage was performed on intravaginally infected mice by flushing the vaginal vault with 2×60 µl Hanks buffered salt solution (HBSS; Trace Scientific, Noble Park, NSW). DNA extractions were performed and Cmu numbers (IFU) determined in whole lungs or vaginal lavage by real-time quantitative PCR and comparison with known standards as previously described [37], [38], [60], [63].

### Airway inflammation

BALF was obtained by cannulation of the trachea and flushing the airways with 2× 1 ml HBSS [37]. BALF cytospins were stained with May-Grunwald-Giemsa and leukocytes enumerated by morphological criteria (≈300 cells by light microscopy [40X]) [37]. All samples were coded and counts performed in a blinded fashion.

### Gene expression analysis

Cytokine expression was evaluated by real-time PCR [38], [60]. Total RNA was extracted from all samples using TRIZOL according to the manufacturer's instructions (Invitrogen, Mount Waverley, VIC). Reverse transcription of RNA (1000 ng) was performed using Superscript III and random hexamer primers (Invitrogen). Relative abundance of genes was determined compared to the reference gene hypoxanthine-guanine phosphoribosyltransferase using a Prism7000 Sequence Detection System (Applied Biosystems, Scoresby, VIC). Primers used were; IFN-γ, Fwd 5′- TCT TGA AAG ACA ATC AGG CCA TCA, Rev 3′-, GAA TCA GCA GCG ACT CCT TTT CC, IL-10, Fwd 5′- CAT TTG AAT TCC CTG GGT GAG AAG, Rev 3′-, GCC TTG TAG ACA CCT TGG TCT TGG, IL-13 Fwd 5′- AGC TGA GCA ACA TCA CAC AAG ACC, Rev 3′-, TGG GCT ACT TCG ATT TTG GTA TCG, 16S of Cmu, Fwd 5′- GCG GCA GAA ATG TCG TTT T, Rev 3′-, CGC TCG TTG CGG GAC TTA and hypoxanthine-guanine phosphoribosyltransferase, Fwd 5′- AGG CCA GAC TTT GTT GGA TTT GAA, Rev 5′- CAA CTT GCG CTC ATC TTA GGC TTT.

### CD4+ T-cell depletion

Mice were treated intraperitoneally with 300 µg αCD4 (clone GK1.5) on days -3, -1, 2 and 5 of Cmu lung infection. The effect of T-cell depletion on bacterial recovery was assessed in whole lungs on day 10. Depletion of CD4+ T-cells was confirmed on day 10 by flow cytometry (Figure S1).

### Assessment of *in vitro* uptake of Cmu by BM macrophages

Femurs and tibias of WT and IL-13−/− mice were collected and bone marrow flushed out with complete RPMI (RPMI 1640, 5×10^−5^ M 2-mercaptoethanol, 10% heat-inactivated FCS, 2 mM L-glutamine, 20 mM HEPES, 100 µg/ml penicillin and 100 µg/ml streptomycin). Red blood cells were lysed and cells washed through a 70 µm nylon cell strainer. The cells were plated out at 1×10^5^ cells/ml in 10 mls complete RPMI supplemented with 15 ng/ml rmGM-CSF (Gift from Walter and Eliza Hall Institute [WEHI], Melbourne) and incubated at 37°C 5% CO_2_. On day 3, another 5 ml medium containing 15 ng/ml rmGM-CSF was added. On day 7, supernatants were removed and non-adherent and semi-adherent cells were removed by washing in cold PBS. Adherent cells were recovered by gentle cell scraping and washed using cold PBS supplemented with 2 mM EDTA. These cells were shown to be >90% macrophages by flow cytometry. BM macrophages were plated out (2×10^5^ cells/ml, 96 well plate) and inoculated with CFSE-labelled, UV-inactivated Cmu (MOI 5) and incubated overnight. (37°C, 5% CO_2_) in complete RPMI. Cultures were washed with complete RPMI to remove all free Cmu and the percentage of BM macrophages (F4/80+ cells) that stained positive for Cmu (CFSE+F4/80+ cells) was determined by flow cytometry.

### Assessment of *in vivo* uptake of Cmu by lung macrophages

BALF was collected from mice on days 3 and 5 following respiratory tract infection. BALF cells were incubated on sterile 10 mm round glass coverslips in a 48 well culture plate for 1 h (37°C, 5% CO_2_) in complete RPMI to allow adhesion of macrophages. Coverslips were removed and adhered cells stained using the *Chlamydia* Cel LPS kit (CelLabs, Brookvale, NSW) according to the manufacturers instructions. The percentage of macrophages that stained positive for the presence of *Chlamydia* was determined in each sample (≈300 macrophages assessed by fluorescent microscopy [40X]). All samples were coded and counts performed in a blinded fashion.

### *In vitro* Cmu infection of mouse lung epithelial cells

LA4 cells (3×10^5^) were plated out on sterile 10 mm glass coverslips in a 48 well culture plate and incubated in Iscove's modified Eagle media (500 µl, 10% fetal calf serum) for 24 h in the presence of either 100 µg αIL-13 mAb (R&D Systems) or PBS (untreated control). Confluent cell monolayers were then infected with Cmu (MOI 5), treated again with αIL-13 mAb or PBS and cultured for a further 24 h. Supernatants were collected and IL-13 ELISA performed according to the manufacturer's instructions (R&D Systems). For staining of chlamydial inclusions, coverslips were removed and cells stained with the *Chlamydia* Cel LPS kit. The percentage of Cmu inclusion positive cells was determined for each treatment (average of >10 fields determined at 40× magnification using a fluorescent microscope). To determine 16S Cmu RNA expression RNA was prepared and assayed by real-time PCR as described above.

### Statistical analysis

Results are presented as mean±SEM. Statistical significance of whole data sets was initially confirmed using one-way ANOVA. The Wilcoxon Rank-sum test was used for non-parametric tests (Mann-Whitney test for two independent samples). P<0.05 was considered statistically significant.

## Supporting Information

Figure S1 Depletion of CD4+ T cells in the lungs of WT mice. WT mice were treated intraperitoneally with 300 μg αCD4 (WT + αCD4, clone GK1.5) on days -3, -1, 2 and 5 of *Cmu* lung infection. The percentage of CD4+ T-cells in the lungs of αCD4 treated mice was assessed on day 10 by flow cytometry and compared to sham-treated (WT) controls. Results are presented as mean ± SEM.
(0.02 MB TIF)
